# A Sterol and Spiroditerpenoids from a *Penicillium* sp. Isolated from a Deep Sea Sediment Sample

**DOI:** 10.3390/md10020497

**Published:** 2012-02-20

**Authors:** Yan Li, Dezan Ye, Zongze Shao, Chengbin Cui, Yongsheng Che

**Affiliations:** 1 Beijing Institute of Pharmacology & Toxicology, Beijing 100850, China; Email: liykx001@126.com; 2 Key Laboratory of Marine Biogenetic Resources, The Third Institute of Oceanography, The State Oceanic Administration, Xiamen 361005, China; Email: yedezan@public.xm.fj.cn (D.Y.); shaozz@163.com (Z.S.)

**Keywords:** *Penicillium* sp., deep sea sediment, sterol, spiroditerpenoids, cytotoxicity

## Abstract

A new polyoxygenated sterol, sterolic acid (**1**), three new breviane spiroditerpenoids, breviones I–K (**2**–**4**), and the known breviones (**5**–**8**), were isolated from the crude extract of a *Penicillium* sp. obtained from a deep sea sediment sample that was collected at a depth of 5115 m. The structures of **1**–**4** were elucidated primarily by NMR experiments, and **1** was further confirmed by X-ray crystallography. The absolute configurations of **2** and **3** were deduced by comparison of their CD spectra with those of the model compounds. Compounds **2** and **5** showed significant cytotoxicity against MCF-7 cells, which is comparable to the positive control cisplatin.

## 1. Introduction

**Figure 1:**
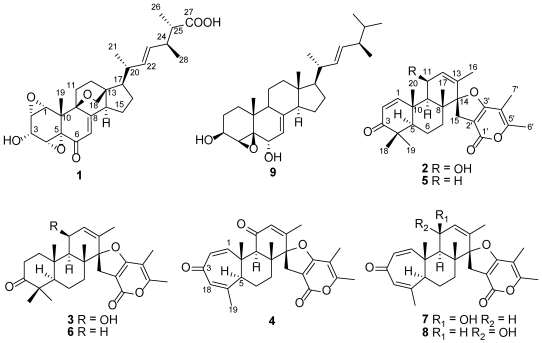
Structures of compounds **1**–**9**.

Marine-derived fungi are recognized as an important source of structurally diverse and pharmacologically active natural products [1,2]. In particular, a growing number of deep sea sediments derived fungi have been reported to produce novel bioactive secondary metabolites [3,4,5,6,7,8,9]. During an ongoing search for new cytotoxic natural products from fungi of unique habitats, we initiated chemical investigations of those fungi isolated from the deep sea sediment samples. In our previous study, we have characterized three new breviane spiroditerpenoids cytotoxic to HeLa Cells from the culture of a *Penicillium* sp. obtained from a deep sea sediment sample that was collected at a depth of 5115 m [8]. Since the crude extract also showed cytotoxicity against two other human tumor cell lines, MCF-7 (breast cancer cells) and A549 (lung carcinoma epithelial cells), and its HPLC fingerprint revealed the presence of minor components that could not be identified. Therefore, the fungus was refermented in a larger scale using the same solid-substrate fermentation medium in which the spiroditerpenoids were first isolated [8]. Fractionation of an EtOAc extract afforded a new polyoxygenated sterol, sterolic acid (**1**), three new breviane spiroditerpenoids, breviones I–K (**2**–**4**), and four known compounds, breviones A (**5**), B (**6**), F (**7**), and G (**8**) (Figure 1) [8,10,11]. Details of the isolation, structure elucidation, and cytotoxicity evaluation of these compounds are reported herein.

## 2. Results and Discussion

**Figure 2:**
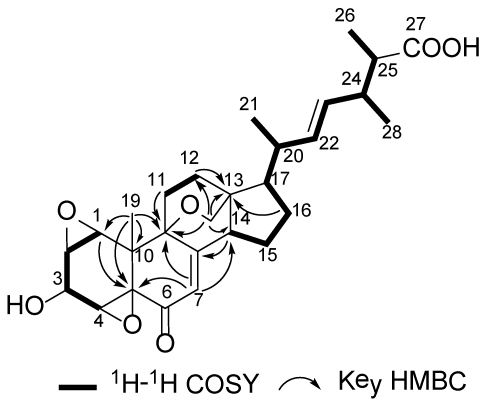
Selected ^1^H–^1^H COSY and HMBC correlations in **1**.

The molecular formula of sterolic acid (**1**) was established as C_28_H_36_O_7_ (11 degrees of unsaturation) on the basis of its HRESIMS (*m/z* = 507.2361 [M + Na]^+^, Δ = −0.8 mmu). Analysis of the ^1^H, ^13^C NMR, and HMQC data (Table 1) of **1** revealed four methyl groups, five methylene units, nine methines including four oxymethines, four sp^3^ quaternary carbons (two of which are oxygenated), four olefinic carbons (three of which are protonated), one α,β-unsaturated ketone carbon (δ_C_ 189.8), and one carboxylic carbon (δ_C_ 180.1), which are characteristic of the C_28_-ergostane-type sterol skeleton. Interpretation of the ^1^H–^1^H COSY NMR data established three spin systems, C-1–C-4, C-11–C-12, and C-14–C-17–C-20–C-28 (Figure 2), which were supported by relevant HMBC correlations. The connectivities of the above mentioned fragments and the remaining functional groups were established on the basis of the key HMBC correlations illustrated in Figure 2, completing the 3-hydroxy-7,22-dien-6-one sterol nucleus. HMBC cross-peaks from H-24, H-25, and H_3_-26 to the C-27 carboxylic carbon (δ_C_ 180.1) connected the carboxyl group to C-25. A key HMBC correlation of H_2_-18 with C-9 revealed an ether linkage between C-18 and C-9 to form an oxabicyclo[2.2.2]octane moiety. Considering the unusual upfield chemical shifts for the oxygenated carbons, C-1 (δ_C_ 58.9), C-2 (δ_C_ 52.6), C-4 (δ_C_ 55.4), and C-5 (δ_C_ 66.2), and the unsaturation requirement for **1**, the presence of two epoxy units was evident. Collectively, these data permitted assignment of the gross structure of **1**.

**Table 1:** NMR data of sterolic acid (**1**) in CDCl_3_.

Position	δ_C_*^a^*, mult.	δ_H_*^b^* (*J* in Hz)	HMBC (H→C#)
1	58.9, CH	3.45, s	2, 5, 10, 19
2	52.6, CH	3.35, s	4
3	64.7, CH	4.25, s	
4	55.4, CH	3.94, s	2, 3
5	66.2, qC		
6	189.8, qC		
7	122.6, CH	5.96, s	5, 9, 14
8	167.8, qC		
9	74.1, qC		
10	38.9, qC		
11a	30.6, CH_2_	1.43, m	
11b		2.51, m	8, 9
12a	30.9, CH_2_	2.29, m	11, 13
12b		2.31, m	13
13	42.8, qC		
14	51.3, CH	2.62, t (9.7)	7, 8, 15, 18
15a	25.1, CH_2_	1.69, m	16
15b		1.97, m	16, 17
16a	29.6, CH_2_	1.54, m	20, 17
16b		2.00, m	13, 15
17	49.8, CH	1.59, m	16, 20
18a	65.6, CH_2_	3.85, d (6.3)	9, 12, 13, 14
18b		3.92, d (6.3)	12, 13, 14
19	19.1, CH_3_	1.15, s	1, 5, 9, 10
20	38.9, CH	2.02, m	16
21	21.4, CH_3_	1.06, d (6.7)	17, 20, 22
22	136.3, CH	5.28, dd (15.0, 8.0)	20, 24
23	130.6, CH	5.19, dd (15.0, 8.0)	20, 24
24	39.6, CH	2.42, q (6.9)	22, 23, 25, 26, 27, 28
25	44.8, CH	2.34, q (6.8)	23, 24, 26, 27, 28
26	14.0, CH_3_	1.10, d (6.8)	24, 25, 27
27	180.1, qC		
28	18.8, CH_3_	1.04, d (6.9)	23, 24, 25

*^a^* Recorded at 100 MHz; *^b^* Recorded at 500 MHz.

**Figure 3:**
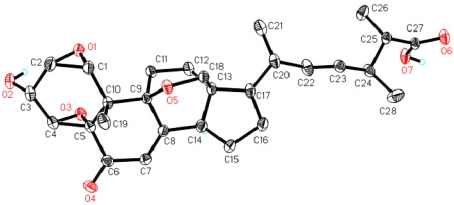
Thermal ellipsoid representation of **1**.

The geometry of the C-22/C-23 olefin was deduced to be *trans* on the basis of the large coupling constant (*J*_22,23_ = 15.0 Hz) observed for the olefinic protons. The relative configuration of other stereogenic centers in **1** was assigned by single crystal X-ray crystallographic analysis (Figure 3). The chemical shift of H_3_-21 (δ_H_ 1.06) supported the 20*R* absolute configuration (H_3_-21 signal appears at1.04 and 0.94 ppm for 20*R* and 20*S* ∆^22^-sterols, respectively) [12,13,14]. Considering the relative configuration established by X-ray data, the absolute configuration of **1** was determined as shown.

**Figure 4:**
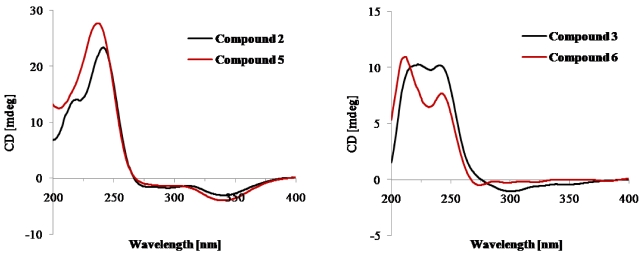
CD spectra of compounds **2**, **3**, **5**, and **6**.

Brevione I (**2**) was assigned the elemental composition C_27_H_34_O_5_ (11 degrees of unsaturation) by HRESIMS (*m/z* 461.2298 [M + Na]^+^; Δ = +0.2 mmu). Analysis of its ^1^H and ^13^C NMR spectroscopic data (Table 2) revealed the presence of one exchangeable proton (δ_H_ 4.01), seven methyl groups, three methylenes, three methines including one oxymethine, four sp^3^ quaternary carbons (one oxygenated), eight olefinic carbons (three of which are protonated), one ester carbonyl carbon (δ_C_ 171.3), and one α,β-conjugated ketone carbon (δ_C_ 203.9). Interpretation of the ^1^H–^1^H COSY and HMBC NMR data of **2** established the gross structure of a spiroditerpenoid, which was the C-11 hydroxylated analogue of the known compound brevione A (**5**), a co-isolated known metabolite which was originally identified from a terrestrial *Penicillium* sp. [10]. The relative configuration of **2** was assigned on the basis of NOESY data and by analogy to **5**. NOESY correlations of H-9 with H-5 and H-11, and of H-5 with H_3_-19 indicated that these protons are all on the same face of the ring system, whereas those of H_3_-17 with H_2_-15 and H_3_-20, and of H_3_-18 with H_3_-20 were used to place them on the opposite face of the molecule, thereby establishing the relative configuration of **2**. The CD spectra of **2** and **5** were nearly identical (Figure 4), suggesting the same absolute configuration for both compounds.

**Table 2:** ^1^H and ^13^C NMR data of breviones I–K (**2**–**4**) in Acetone-*d*_6_.

	Breviones I (2)			Breviones J (3)			Breviones K (4)	
Position	δ_C_*^a^*, mult.	δ_H_*^b^* (*J* in Hz)		δ_C_*^c^*, mult.	δ_H_*^b^* (*J* in Hz)		δ_C_*^c^*, mult.	δ_H_*^b^* (*J* in Hz)
1a	158.4, CH	7.50, d (11)		38.4, CH_2_	2.29, ddd (16, 6.0, 3.2)		155.2, CH	6.83, d (13)
1b					2.34, ddd (16, 6.0, 3.2)			
2a	125.6, CH	5.80, d (11)		33.8, CH_2_	2.69, ddd (16, 6.0, 3.2)		128.4, CH	5.70, dd (13, 2.1)
2b					2.74, ddd (16, 6.0, 3.2)			
3	203.9, qC			215.1, qC			192.5, qC	
4	44.8, qC			50.8, qC			154.0, qC	
5	54.6, CH	1.63, m		56.3, CH	1.75, m		48.6, CH	2.93, d (13)
6a	19.3, CH_2_	1.64, m		18.8, CH_2_	1.63, m		21.7, CH_2_	1.83, dt (13, 3.5)
6b		1.80, m			1.82, m			1.93, td (13, 3.5)
7a	33.0, CH_2_	1.50, m		32.3, CH_2_	1.48, m		30.3, CH_2_	1.65, td (13, 3.5)
7b		1.62, m			1.56, m			1.74, dt (13, 3.5)
8	41.9, qC			40.8, qC			45.4, qC	
9	47.1, CH	1.95, d (4.5)		47.2, CH	1.91, br		54.8, CH	3.15, s
10	41.3, qC			40.2, qC			42.0, qC	
11	64.4, CH	4.78, s		64.1, CH	4.51, s		198.2, qC	
12	131.3, CH	5.79, s		131.0, CH	5.72, d (5.0)		130.2, CH	5.91, s
13	133.1, qC			132.2, qC			151.8, qC	
14	99.8, qC			99.1, qC			97.7, qC	
15a	29.5, CH_2_	2.96, d (16)		29.7, CH_2_	2.94, d (16)		30.1, CH_2_	3.15, s
15b		3.01, d (16)			2.97, d (16)			
16	18.8, CH_3_	1.74, s		18.2, CH_3_	1.71, s		18.5, CH_3_	1.97, s
17	20.0, CH_3_	1.32, s		16.7, CH_3_	1.29, s		18.3, CH_3_	1.18, s
18	27.8, CH_3_	1.08, s		26.0, CH_3_	1.05, s		131.7, CH	5.99, s
19	22.2, CH_3_	1.06, s		21.4, CH_3_	1.01, s		24.1, CH_3_	1.99, s
20	21.5, CH_3_	1.67, s		19.4, CH_3_	1.59, s		14.3, CH_3_	1.41, s
1'	171.3, qC			170.6, qC			170.8, qC	
2'	99.8, qC			99.3, qC			100.1, qC	
3'	161.2, qC			159.4, qC			161.8, qC	
4'	103.0, qC			102.4, qC			103.2, qC	
5'	161.0, qC			160.4, qC			160.7, qC	
6'	9.5, CH_3_	1.85, s		8.7, CH_3_	1.86, s		9.5, CH_3_	1.92, s
7'	17.1, CH_3_	2.17, s		16.4, CH_3_	2.17, s		17.2, CH_3_	2.20, s
OH-11		4.01, br			3.72, d (5.0)			

*^a^* Recorded at 100 MHz; *^b^* Recorded at 500 MHz; *^c^* Recorded at 150 MHz.

Brevione J (**3**) gave a pseudomolecular ion [M + Na]^+^ peak at *m/z* = 463.2455 (Δ = +0.7 mmu) by HRESIMS, consistent with an elemental composition of C_27_H_36_O_5_ (10 degrees of unsaturation). Analysis of its NMR data (Table 2) revealed the presence of similar structural features as those found in **2**, except that the C-1/C-2 olefin was replaced by two mutually-coupled methylenes, and this observation was supported by relevant ^1^H–^1^H COSY and HMBC correlations. Therefore, the planar structure of **3** was proposed as shown. NOESY correlations of H-5 with H-9 and H_3_-19, and of H-9 with H-11 indicated that these protons are all on the same face of the ring system, whereas those of H_3_-17 with H-6b, H_2_-15 and H_3_-20, and of H-6b with H_3_-18 were used to place them on the opposite face of the molecule, thereby establishing the relative configuration of **3**. The absolute configuration of **3** was deduced to be the same as that of the co-isolated known compound **6** [11] by comparison of their NMR and CD data (Figure 4).

Brevione K (**4**) was isolated as a yellow powder with a molecular formula of C_27_H_30_O_5_ (13 degrees of unsaturation), established by HRESIMS (*m/z* 457.1985 [M + Na]^+^; Δ = +0.5 mmu). The ^1^H and ^13^C NMR spectra of **4** (Table 2) showed resonances similar to those of the co-isolated known compounds, breviones F (**7**) and G (**8**) [8], except that the C-11 oxymethine was replaced by an α,β-conjugated ketone carbon (δ_C_ 198.2), which was confirmed by an HMBC correlation from H-9 to C-11. The relative configuration of **4** was assigned on the basis of NOESY data and by analogy to **7** and **8**. A NOESY correlation of H-9 with H-5 placed the two protons on the same face of the ring system, whereas those of H_3_-17 with H_2_-15 and H_3_-20 indicated that these protons were on the opposite face, establishing the relative configuration of **4**. The absolute configuration of **4** was deduced by semisynthetic method [15]. Specifically, treatment of **7** and **8** with manganese dioxide both afforded an oxidation product of OH-11, and the ^1^H NMR data and specific rotation value of the product were identical to **4**, suggesting the 5*S*, 8*R*, 9*R*, 10*S*, and 14*S* absolute configuration.

Compounds **1**–**8** were tested for cytotoxicity against two human tumor cell lines, MCF-7 and A549. Compounds **2** and **5** showed cytotoxic effects against MCF-7 cells, with IC_50_ values of 7.44 and 28.4 µM, respectively, whereas **2** also displayed activity against A549 cells, with an IC_50_ value of 32.5 µM (the positive control cisplatin showed IC_50_ values of 8.09 and 8.90 µM, respectively, against the two tumor cell lines). Other compounds did not show detectable cytotoxicity against the two cell lines at 50 µg/mL.

## 3. Experimental Section

### 3.1. General Experimental Procedures

Optical rotations were measured on a Perkin-Elmer 241 polarimeter, and UV data were recorded on a Shimadzu Biospec-1601 spectrophotometer. CD spectra were recorded on a JASCO J-815 spectropolarimeter. IR data were recorded using a Nicolet Magna-IR 750 spectrophotometer. ^1^H and ^13^C NMR data were acquired with Varian Mercury-400, Inova-500 and NMR system-600 spectrometers using solvent signals (acetone-*d*_6_: δ_H_ 2.05/δ_C_ 29.8, 206.1; CDCl_3_: δ_H_ 7.26/δ_C_ 76.7) as references. The HMQC and HMBC experiments were optimized for 145.0 and 8.0 Hz, respectively. ESIMS data were recorded on a Bruker Esquire 3000^plus^ spectrometer, and HRESIMS data were obtained using Bruker APEX III 7.0 T and APEX II FT-ICR spectrometers, respectively.

### 3.2. Fungal Material

The *Penicillium* sp. was isolated by one of the authors (D.Y.) from a deep water sediment sample collected at a depth of 5115 m in the East Pacific Ocean (145°2′W, 07°37′N), in September 2003. The isolate was characterized as an unidentified species of *Penicillium* by one of authors (Z.S.) based on sequence (Genebank accession number EU139854) analysis of the ITS region of the rDNA and assigned the accession number 3A00005 in the Marine Culture Collection Center (MCCC) at the Third Institute of Oceanography, the State Oceanic Administration, Xiamen, People’s Republic of China. The fungal strain was cultured on slants of potato dextrose agar (PDA) with artificial seawater (NaCl 23.5 g, MgCl_2_·6H_2_O 10.6 g, CaCl_2_·2H_2_O 1.5 g, KCl 0.66 g, Na_2_SO_4_ 3.9 g, NaHCO_3_ 0.2 g, H_3_BO_3_ 0.03 g in 1 L distilled H_2_O) at 25 °C for 7 days. Agar plugs were cut into small pieces (about 0.5 × 0.5 × 0.5 cm^3^) under aseptic conditions, 15 pieces were used to inoculate in three Erlenmeyer flasks (250 mL), each containing 50 mL of media (0.4% glucose, 1% malt extract, and 0.4% yeast extract in artificial seawater); the final pH of the media was adjusted to 6.5 and sterilized by autoclave. Three flasks of the inoculated media were incubated at 25 °C on a rotary shaker at 170 rpm for five days to prepare the seed culture. Fermentation was carried out in 12 Fernbach flasks (500 mL), each containing 80 g of rice. Spore inoculum was prepared by suspension in sterile, distilled H_2_O to give a final spore/cell suspension of 1 × 10^6^/mL. Artificial seawater (120 mL) was added to each flask, and the contents were soaked overnight before autoclaving at 15 psi for 30 min. After cooling to room temperature, each flask was inoculated with 5.0 mL of the spore inoculum and incubated at 25 °C for 40 days.

### 3.3. Extraction and Isolation

The fermented material was extracted repeatedly with EtOAc (4 × 1.0 L), and the organic solvent was evaporated to dryness under vacuum to afford the crude extract (7.5 g), which was fractionated by silica gel VLC using petroleum ether-EtOAc gradient elution. The fractions eluted with 40 (125 mg) and 45% (65 mg) EtOAc were combined and separated again by Sephadex LH-20 column chromatography (CC) using 1:1 CH_2_Cl_2_-MeOH as eluents, and the resulting subfractions were combined and further purified by semipreparative RP HPLC (Agilent Zorbax SB-C_18_ column; 5 μm; 9.4 × 250 mm; 43% CH_3_CN in H_2_O for 40 min; 2 mL/min) to afford breviones I (**2**; 5.2 mg, *t*_R_ 37.22 min) and J (**3**; 3.6 mg, *t*_R_ 38.81 min). The fraction (86 mg) eluted with 100% EtOAc was fractionated again by Sephadex LH-20 CC eluting with MeOH as eluents. One subfraction (28 mg) was further purified by RP HPLC (Agilent Zorbax SB-C_18_ column; 5 μm; 9.4 × 250 mm; 65% MeOH in H_2_O for 15 min followed by 65–100% for 20 min; 2 mL/min) to afford sterolic acid (**1**; 6.0 mg, *t*_R_ = 28.60 min). The fraction (120 mg) eluted with 15% EtOAc was separated again by Sephadex LH-20 CC eluting with 1:1 CH_2_Cl_2_-MeOH. The resulting subfractions were combined and further purified by RP HPLC (85% MeOH in H_2_O for 25 min 2 mL/min) to afford **5** (18.0 mg, *t*_R_ 16.04 min) and **6** (14.5 mg, *t*_R_ 18.51 min). The fractions eluted with 50 (163 mg) and 60% (225 mg) EtOAc were combined and fractionated again by Sephadex LH-20 CC using 1:1 CH_2_Cl_2_-MeOH. Purification of the resulted subfractions with different isocratic elutions by RP HPLC afforded breviones K (**4**; 4.8 mg, *t*_R_ 41.9 min; 65% CH_3_OH in H_2_O for 45 min), F (**7**; 9.5 mg, *t*_R_ 54.38 min; 58% CH_3_OH in H_2_O for 60 min), and G (**8**; 12.5 mg, *t*_R_ 44.71 min; same elution as in purification of **7**), respectively.

**Sterolic** **acid (1):** yellow powder; [*α*]^25^_D_−25 (*c* 0.1, MeOH); UV (MeOH) λ_max_ (log ε) 202 (2.13), 246 (2.31) nm; IR (neat) *ν*_max_ 3379 (br), 2962, 2931, 2874, 1693, 1682, 1624, 1154, 1477, 1254, 1215, 1057, 972 cm^−1^; ^1^H, ^13^C NMR, and HMBC data see Table 1; HRESIMS *m/z* 507.2361 [M + Na]^+^ (calcd. for C_2__8_H_3__6_O_7_Na, 507.2353).

**Brevione** **I (2):** white powder; [*α*]^25^_D_ +96 (*c* 0.1, CHCl_3_); UV (CH_3_OH) λ_max_ (log ε) 212 (4.01), 296 (2.28) nm; CD (*c* 4.6 × 10^−^^4^ M, MeOH) λ_max_ (Δε) 219 (+14.05), 241 (+23.34), 294 (−1.73), 341 (−3.03); IR (neat) *ν*_max_ 3436 (br), 2947, 1701, 1670, 1574, 1445, 1390, 1275, 1061, 984 cm^−1^; ^1^H and ^13^C NMR data, see Table 2; HMBC data (acetone-*d*_6_, 400 MHz) H-1→C-3, 5, 9, 10; H-2→C-4, 10; H-5→C-1, 2, 7, 9, 18, 19, 20; H-6a→C-4, 10; H-6b→C-5; H-7a→C-5, 8, 14, 17; H-7b→C-6, 9; H-9→C-1, 5, 8, 10, 11, 12, 14, 17, 20; H-11→C-8, 9, 12, 13; H-12→C-9, 14; H-15a→C-8, 13, 14, 1', 3'; H-15b→C-8, 13, 14, 1', 3'; H_3_-16→C-12, 13, 14; H_3_-17→C-7, 8, 9, 14; H-18→C-3, 4, 5, 19; H_3_-19→C-3, 4, 5, 18; H_3_-20→C-1, 5, 9, 10; H_3_-6'→C-1', 4', 5'; H_3_-7'→C-3', 4', 5'; NOESY correlations (acetone-*d*_6_, 400 MHz) H-1↔H-9, H-11, H_3_-20; H-2↔H-11; H-5↔H-9, H_3_-19; H-6a↔H-9, H_3_-19; H-6b↔H_3_-17, H_3_-18, H_3_-20; H-7a↔H-9; H-7b↔H-15b, H_3_-17; H-9↔H-1, H-5, H-6a, H-7a, H-11; H-11↔H-1, H-2, H-9; H-12↔H_3_-16; H-15a↔H_3_-16, H_3_-17; H-15b↔H-7b, H_3_-17; H_3_-16↔H-12, H-15a; H_3_-17↔H-6b, H-7b, H-15a, H-15b, H_3_-20; H_3_-18↔H-6b; H_3_-19↔H-5, H-6a; H_3_-20↔H-1, H-6b, H_3_-17; H_3_-6'↔H_3_-7'; HRESIMS *m/z* 461.2298 [M + Na]^+^ (calcd. for C_27_H_34_O_5_Na, 461.2300).

**Brevione** **J (3):** white powder; [*α*]^25^_D_ +64 (*c* 0.1, CHCl_3_); UV (CH_3_OH) λ_max_ (log ε) 212 (2.96), 244 (3.38), 297 (3.30) nm; CD (*c* 4.6 × 10^−^^4^ M, MeOH) λ_max_ (Δε) 212 (+9.22), 244 (+10.03), 297 (−1.01); IR (neat) *ν*_max_ 3494 (br), 2951, 1704, 1651, 1574, 1444, 1389, 1275, 1062, 983 cm^−1^; ^1^H and ^13^C NMR data, see Table 2; NOESY correlations (acetone-*d*_6_, 600 MHz) H-1a↔H-5, H-9, H-11; H-1b↔H_3_-20; H-2a↔H-11; H-2b↔H_3_-20; H-5↔H-1a, H-9, H_3_-19; H-6a↔H-9, H_3_-19; H-6b↔H_3_-17, H_3_-18, H_3_-20; H-7a↔H-9; H-7b↔H-15b, H_3_-17; H-9↔H-1a, H-5, H-6a, H-7a, H-11; H-11↔H-1a, H-2a, H-9; H-12↔H_3_-16; H-15a↔H_3_-16, H_3_-17; H-15b↔H-7b, H_3_-17; H_3_-16↔H-12, H-15a; H_3_-17↔H-6b, H-7b, H-15a, H-15b, H_3_-20; H_3_-18↔H-6b; H_3_-19↔H-5, H-6a; H_3_-20↔H-1b, H-2b, H-6b, H_3_-17; H_3_-6'↔H_3_-7'; HRESIMS *m/z* 463.2455 [M + Na]^+^ (calcd. for C_27_H_36_O_5_Na, 463.2462).

**Brevione** **K (4):** yellow powder; [*α*]^25^_D_ +34 (*c* 0.2, CHCl_3_); UV (CH_3_OH) λ*_m_*_ax_ (log ε) 208 (3.60), 234 (3.51), 294 (2.95) nm; IR (neat) *ν*_max_ 2934, 1724, 1668, 1652, 1624, 1582, 1440, 1393, 1273, 1113 cm^−1^; ^1^H and ^13^C NMR data, see Table 2; HMBC data (acetone-*d*_6_, 600 MHz) H-1→C-3, 5, 9, 10; H-2→C-10, 18; H-6a→C-5, 8; H-6b→C-5, 7; H-7a→C-6, 17; H-7b→C-5, 6, 8, 17; H-9→C-1, 5, 10, 11, 17, 20; H-12→C-9, 14, 16; H_2_-15→C-8, 13, 14, 1', 2', 3', 4'; H_3_-16→C-12, 13, 14; H_3_-17→C-7, 8, 9, 14; H-18→C-2, 5, 19; H_3_-19→C-4, 5, 18; H_3_-20→C-1, 5, 9, 10; H_3_-6'→C-1', 4', 5'; H_3_-7'→C-4', 5'; NOESY correlations (acetone-*d*_6_, 600 MHz) H-1↔H-9; H-5↔H-9; H-6b↔H_2_-15, H_3_-17, H_3_-20; H-7b↔H_2_-15, H_3_-17; H-9↔H-1, H-5; H-12↔H_3_-16; H_2_-15↔H-6b, H-7b, H_3_-16, H_3_-17; H_3_-16↔H-12, H_2_-15; H_3_-17↔H-6b, H-7b, H_2_-15, H_3_-20; H-18↔H_3_-19; H_3_-20↔H-6b, H_3_-17; H_3_-6'↔H_3_-7'; HRESIMS *m/z* 457.1985 [M + Na]^+^ (calcd. for C_27_H_30_O_5_Na, 457.1990).

**Brevione** **A (5):** white powder; CD (*c* 4.6 × 10^−^^4^ M, MeOH) λ_max_ (Δε) 237 (+27.7), 294 (−1.38), 341 (−3.93); ^1^H, ^13^C NMR, and the ESIMS data were fully consistent with literature [10].

**Brevione** **B (6):** white powder; CD (*c* 4.6 × 10^−^^4^ M, MeOH) λ_max_ (Δε) 212 (+10.96), 242 (+7.69), 274 (−0.50); ^1^H, ^13^C NMR, and the ESIMS data were fully consistent with literature [11].

**Brevione** **F (7):**^1^H, ^13^C NMR, and the ESIMS data were fully consistent with literature [8].

**Brevione** **G (8):**^1^H, ^13^C NMR, and the ESIMS data were fully consistent with literature [8].

**Oxidation of** **brevione F (7)** **to** **brevione** **K(4):** A solution of brevione F (**7**; 1.5 mg, 0.034 mmol) in dry benzene (1.0 mL) was treated with MnO_2_ (3.0 mg, 0.034 mmol). The mixture was stirred at 25 °C for 10 days, filtered, and washed with diethyl ether. The filtrate was concentrated under reduced pressure and the residue was purified by RP HPLC (Agilent Eclipse plus C_18_ column; 3.5 μm; 4.6 × 100 mm; 20% MeOH in H_2_O for 1 min, followed by 20–100% over 15 min; 1 mL/min) to afford **4** (0.6 mg, *t*_R_ 12.88 min, 40% yield).

**Oxidation of** **brevione G (8)** **to** **brevione** **K(4):** A solution of brevione G (**8**; 1.5 mg, 0.034 mmol) in dry benzene (1.0 mL) was treated with MnO_2_ (3.0 mg, 0.034 mmol). The mixture was stirred at 25 °C for 10 days, filtered, and MnO_2_ was washed with diethyl ether. The filtrate was concentrated under reduced pressure and the residue was purified by RP HPLC (the same HPLC conditions as above) to afford **4** (0.5 mg, *t*_R_ 12.88 min, 33% yield).

### 3.4. X-ray Crystallographic Analysis of ***1***

Upon crystallization from MeOH/H_2_O (10:1) using the vapor diffusion method, colorless crystals were obtained for **1**, and a crystal (0.33 × 0.23 × 0.07 mm) was separated from the sample and mounted on a glass fiber, and data were collected using a Rigaku Saturn CCD area detector with graphite-monochromated Mo Kα radiation, λ = 0.71073 Ǻ at 173(2) K. Crystal data: C_28_H_3__6_O_7_, *M* = 484.57, space group orthorhombic, *P*2_1_2_1_2_1_; unit cell dimensions *a* = 9.6480 (19) Å, *b* = 14.209 (3) Å, *c* = 18.550 (4) Å, *V* = 2543.0 (9) Å^3^, *Z* = 4, *D*_calcd_ = 1.266 mg/m^3^, μ = 0.090 mm^−1^, *F*(000) = 1040. The structure was solved by direct methods using SHELXL-97 [16] and refined using full-matrix least-squares difference Fourier techniques. All non-hydrogen atoms were refined with anisotropic displacement parameters, and all hydrogen atoms were placed in idealized positions and refined as riding atoms with the relative isotropic parameters. Absorption corrections were applied with the Siemens Area Detector Absorption Program (SADABS) [17]. The 2,5647 measurements yielded 5825 independent reflections after equivalent data were averaged, and Lorentz and polarization corrections were applied. The final refinement gave *R_1_* = 0.0414 and *wR_2_* = 0.0303 [*I* > 2σ(*I*)]. Crystallographic data for compound **1** have been deposited with the Cambridge Crystallographic Data Centre as supplementary publication number CCDC 859857. Copies of the data can be obtained, free of charge, on application to the director, CCDC 12 Union Road, Cambridge CB2 1EZ, UK [18].

### 3.5. MTS Assay

The assay was run in triplicate. In a 96-well plate, each well was plated with (2–5) × 10^3^ cells (depending on the cell multiplication rate). After cell attachment overnight, the medium was removed, and each well was treated with 100 µL medium containing 0.1% DMSO, or appropriate concentrations of the test compounds and the positive control cisplatin (100 mM as stock solution of a compound in DMSO and serial dilutions; the test compounds showed good solubility in DMSO and did not precipitate when added to the cells). The plate was incubated for 48 h at 37 °C in a humidified, 5% CO_2_ atmosphere. Proliferation assessed by adding 20 μL of MTS (Promega) to each well in the dark, followed by a 90 min incubation at 37 °C. The assay plate was read at 490 nm using a microplate reader [19].

## 4. Conclusions

Sterolic acid (**1**) is the first example of the sterols possessing the unusual 1,2:4,5-diepoxy, oxabicyclo[2.2.2]octane with a C-27 carboxylic group. Although the polyoxygenated sterols were encountered frequently in natural products, the diepoxy sterols are relatively rare, with only three marine-derived 5,6:8,9-diepoxy sterols as the reported precedents [20]. Sterols containing the oxabicyclo[2.2.2]octane moiety are also rare, only the plant metabolite 12β,14β-dihydroxy-3β,19-epoxy-3a-methoxy-5a-card-20(22)-enolide has been reported to date [21]. Structurally, **1** is related to the known compound gargalol A (**9**), a sterol isolated from an edible mushroom *Grifola gargal* [22]. However, **1** differs significantly from **9** by having more complexed structural features. Breviones I–K (**2**–**4**) are new members of the breviane spiroditerpenoid class of metabolites with mixed biogenesis [8,10,11]. Compounds **2** and **3** are closely related to the known breviones A (**5**) and B (**6**) [10,11], respectively, but differ in having a hydroxy group at C-11, whereas compound **4** has a ketone group at C-11 compared to the co-isolated known compounds **7** and **8** [8]. To date, total syntheses of several breviane spiroditerpenoids have been achieved [23,24,25,26,27] including the enantioselective synthesis of breviones A–C [27]. In the current work, the isolation of additional new secondary metabolites, especially the structurally unique sterol demonstrated that the marine-derived fungi from deep water sediments deserve increased attention.

## Supplementary Files

Supplementary File 1: ZIP-Document (ZIP, 299 KB)

## References

[1] Blunt J.W., Copp B.R., Munro M.H.G., Northcote P.T., Prinsep M.R. (2004). Marine natural products. Nat. Prod. Rep..

[2] Blunt J.W., Copp B.R., Munro M.H.G., Northcote P.T., Prinsep M.R. (2011). Marine natural products. Nat. Prod. Rep..

[3] Gautschi J.T., Amagata T., Amagata A., Valeriote F.A., Mooberry S.L., Crews P. (2004). Expanding the strategies in natural product studies of marine-derived fungi: A chemical investigation of *Penicillium* obtained from deep water sediment. J. Nat. Prod..

[4] Lebar M.D., Heimbegnier J.L., Baker B.J. (2007). Cold-water marine natural products. Nat. Prod. Rep..

[5] Li D.H., Wang F.P., Xiao X., Fang Y.C., Zhu T.J., Gu Q.Q., Zhu W.M. (2007). Trisorbicillinone A, a novel sorbicillin trimer, from a deep sea fungus, *Phialocephala* sp. FL30r. Tetrahedron Lett..

[6] Skropeta D. (2008). Deep-sea natural products. Nat. Prod. Rep..

[7] Wilson Z.E., Brimble M.A. (2009). Molecule derived from the extremes of life. Nat. Prod. Rep..

[8] Li Y., Ye D.Z., Chen X.L., Lu X.H., Shao Z.Z., Zhang H., Che Y.S. (2009). Breviane spiroditerpenoids from an extreme-tolerant *Penicillium* sp. isolated from a deep sea sediment sample. J. Nat. Prod..

[9] Li D.H., Cai S.X., Zhu T.J., Wang F.P., Xiao X., Gu Q.Q. (2010). Three new sorbicillin trimers, trisorbicillinones B, C, and D, from a deep ocean sediment derived fungus, *Phialocephala* sp. FL30r. Tetrahedron.

[10] Macias F.A., Varela R.M., Simonet A.M., Cutler H.G., Cutler S.J., Ross S.A., Dunbar D.C., Dugan F.M., Hill R.A. (2000). (+)-Brevione A. The first member of a novel family of bioactive spiroditerpenoids isolated from *Penicillium brevicompactum* Dierckx. Tetrahedron Lett..

[11] Macias F.A., Varela R.M., Simonet A.M., Cutler H.G., Cutler S.J., Dugan F.M., Hill R.A. (2000). Novel bioactive breviane spiroditerpenoids from *Penicillium brevicompactum* Dierckx. J. Org. Chem..

[12] Vanderah D.J., Djerassi C. (1978). Marine natural products. Synthesis of four naturally occurring 20.beta.-H cholanic acid derivatives. J. Org. Chem..

[13] Iorizzi M., Minale L., Riccio R., Debray M., Menou J.L. (1986). Starfish saponins, part 23. Steroidal glycosides from the starfish *Halityle regularis*. J. Nat. Prod..

[14] Mansoor T.A., Hong J., Lee C.O., Bae S.J., Im K.S., Jung J.H. (2005). Cytotoxic sterol derivatives from a marine sponge *Homaxinella* sp.. J. Nat. Prod..

[15] Krohn K., Kouam S.F., Kuigoua G.M., Hussain H., Flörke S.C.-B.U., Kurtán T., Pescitelli G., Di Bari L., Draeger S., Schulz B. (2009). Xanthones and oxepino[2, 3-b]chromones from three endophytic fungi. Chem. Eur. J..

[16] (1997). *SHELXL-97*, program for x-ray crystal structure solution and refinement.

[17] (1999). *SADABS*, version 2.03; program for empirical absorption correction of area detector data.

[B18-marinedrugs-10-00497] 18.Cambridge Crystallographic Data Centre. Fax: +44-1223-336033; E-Mail: deposit@ccdc.cam.ac.uk.

[19] Zhang N., Chen Y.L., Jiang R.X., Li E.W., Chen X.L., Xi Z.J., Guo Y.L., Liu X.Z., Zhou Y.G., Che Y.S. (2011). PARP and RIP1 are required for autophagy induced by 11'-deoxyverticillin A, which precedes caspase-dependent apoptosis. Autophagy.

[20] Mansoor T.A., Lee Y.M., Hong J., Lee C.O., Im K.S., Jung J.H. (2006). 5,6:8,9-Diepoxy and other cytotoxic sterols from the marine sponge *Homaxinella* sp.. J. Nat. Prod..

[21] Li J.Z., Qing C., Chen C.X., Hao X.J., Liu H.Y. (2009). Cytotoxicity of cardenolides and cardenolide glycosides from *Asclepias curassavica*. Bioorg. Med. Chem. Lett..

[22] Wu J., Choi J.H., Yoshida M., Hirai H., Harada E., Masuda K., Koyama T., Yazawa K., Noguchi K., Nagasawa K. (2011). Osteoclast-forming suppressing compounds, gargalols A, B, and C, from the edible mushroom *Grifola gargal*. Tetrahedron.

[23] Takikawa H., Hirooka M., Sasaki M. (2002). Synthetic studies on breviones: Construction of the CDE ring system. Tetrahedron Lett..

[24] Takikawa H., Hirooka M., Sasaki M. (2003). The first synthesis of (±)-brevione B, an allelopathic agent isolated from *Penicillium* sp.. Tetrahedron Lett..

[25] Takikawa H., Imamura Y., Sasaki M. (2006). Synthesis and absolute configuration of brevione B, an allelochemical isolated from *Penicillium* sp.. Tetrahedron.

[26] Macías F.A., Carrera C., Chinchilla N., Fronczek F.R., Galindo J.C. (2010). Synthesis of the western half of breviones C, D, F and G. Tetrahedron.

[27] Yokoe H., Mitsuhashi C., Matsuoka Y., Yoshimura T., Yoshida M., Shishido K. (2011). Enantiocontrolled total syntheses of breviones A, B, and C. J. Am. Chem. Soc..

