# Supplementary material for: A Sterol and Spiroditerpenoids from a Penicillium sp. Isolated from a Deep Sea Sediment Sample
Source: Mar Drugs. 2012 Feb 20;10(2):497–508. doi: 10.3390/md10020497 (PMC3297011; doi:10.3390/md10020497)

Supplementary Materials

Contents **Page**

1) **Figure S1.** ^{1}H NMR spectrum of sterolic acid (**1**; 500 MHz, $CDCl_3$) 2

2) **Figure S2.** ^{13}C NMR spectrum of sterolic acid (**1**; 100 MHz, $CDCl_3$) 3

3) **Figure S3.** ^{1}H NMR spectrum of brevione I (**2**; 500 MHz, acetone-d_6) 4

4) **Figure S4.** ^{13}C NMR spectrum of brevione I (**2**; 100 MHz, acetone-d_6) 5

5) **Figure S5.** NOESY spectrum of brevione I (**2**; 600 MHz, acetone-d_6) 6

6) **Figure S6.** ^{1}H NMR spectrum of brevione J (**3**; 500 MHz, acetone-d_6) 7

7) **Figure S7.** ^{13}C NMR spectrum of brevione J (**3**; 100 MHz, acetone-d_6) 8

8) **Figure S8.** NOESY spectrum of brevione J (**3**; 600 MHz, acetone-d_6) 9

9) **Figure S9.** ^{1}H NMR spectrum of brevione K (**4**; 500 MHz, acetone-d_6) 10

10) **Figure S10.** ^{13}C NMR spectrum of brevione K (**4**; 150 MHz, acetone-d_6) 11

11) **Figure S11.** NOESY spectrum of brevione K (**4**; 600 MHz, acetone-d_6) 12

Figure S1. ^{1}H NMR Spectrum of Sterolic Acid (**1**; 500 MHz, $CDCl_3$)

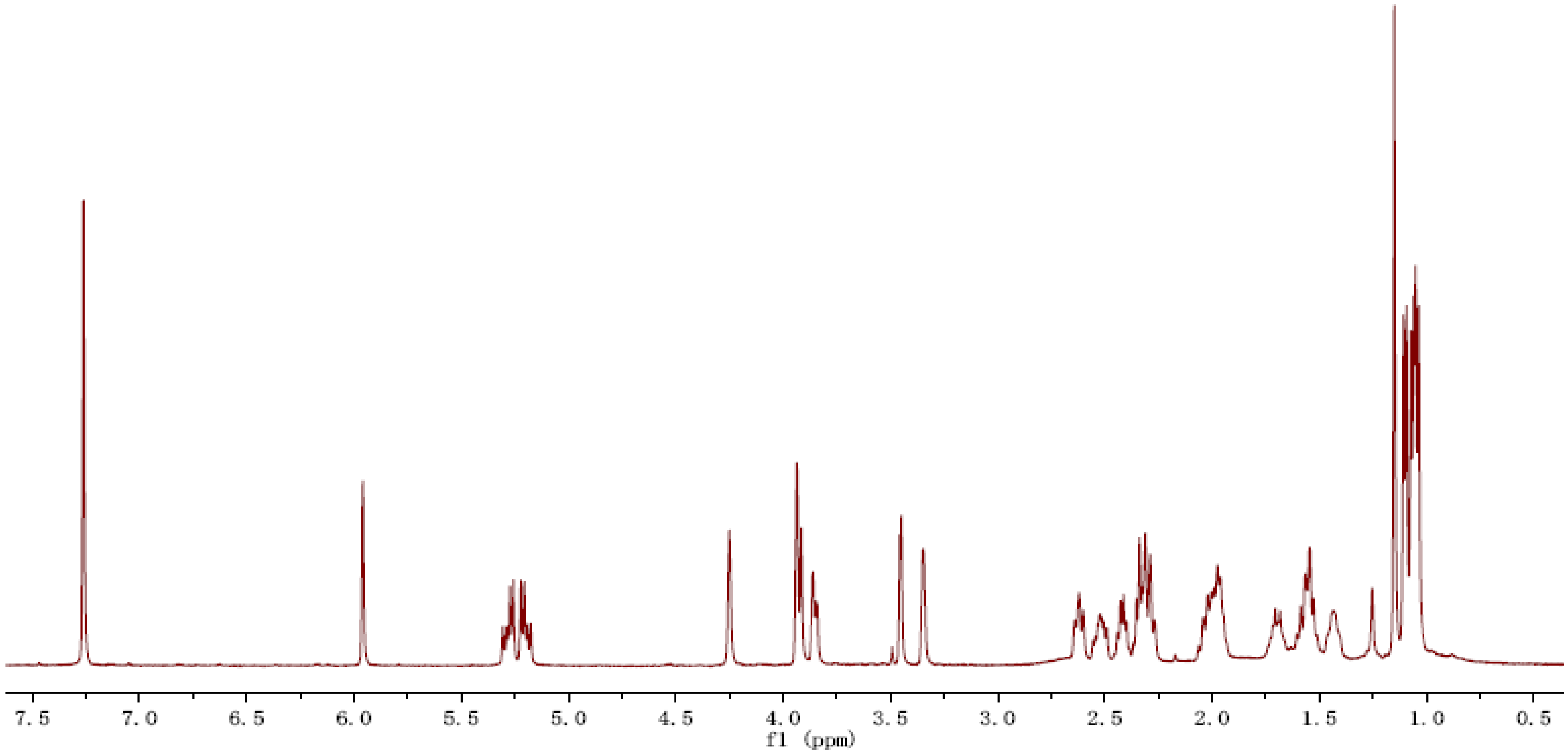

Figure S2. ^{13}C NMR Spectrum of Sterolic Acid (**1**; 100 MHz, $CDCl_3$)

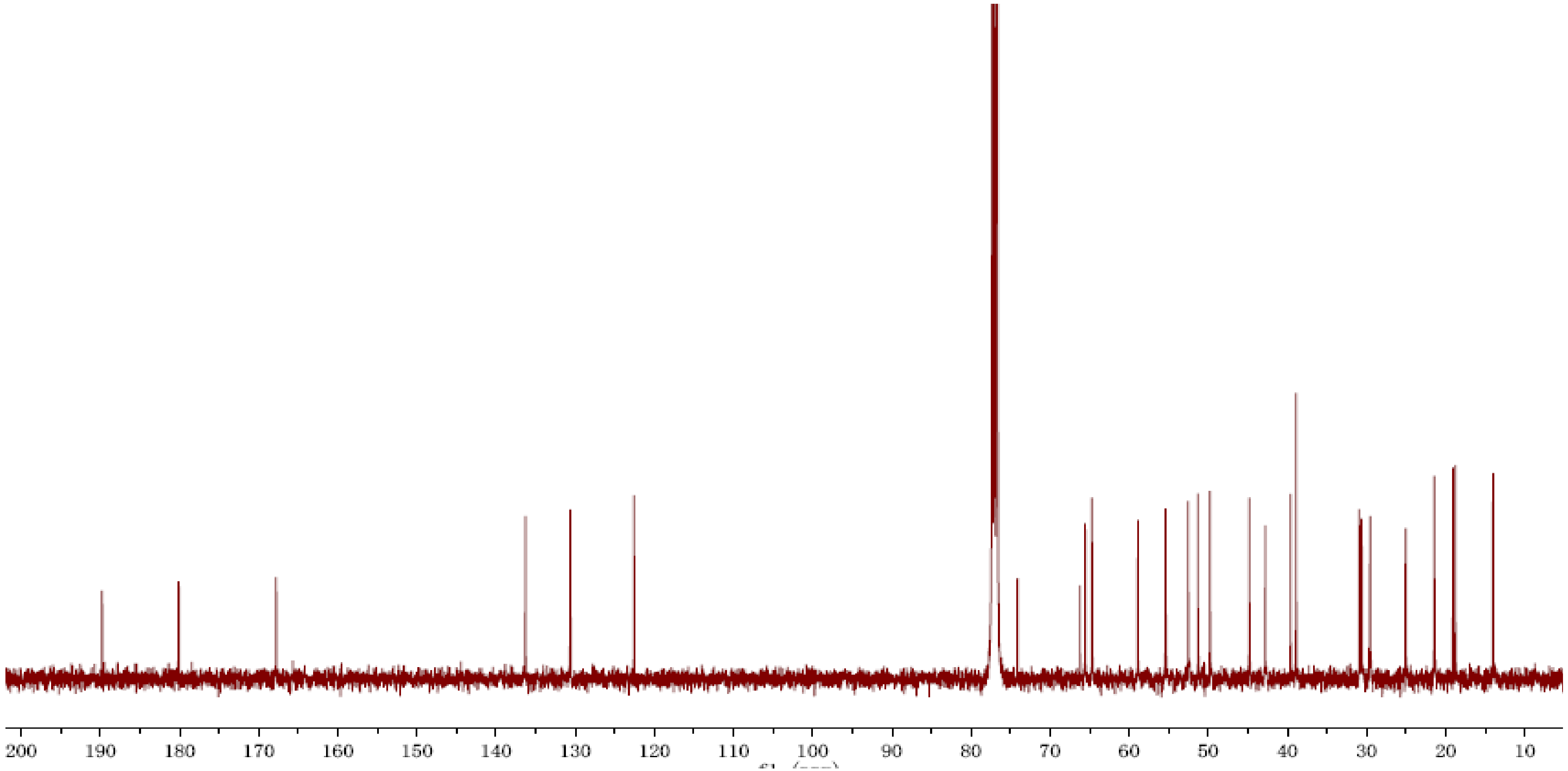

Figure S3. ^{1}H NMR Spectrum of Brevione I (**2**; 500 MHz, Acetone-d_6)

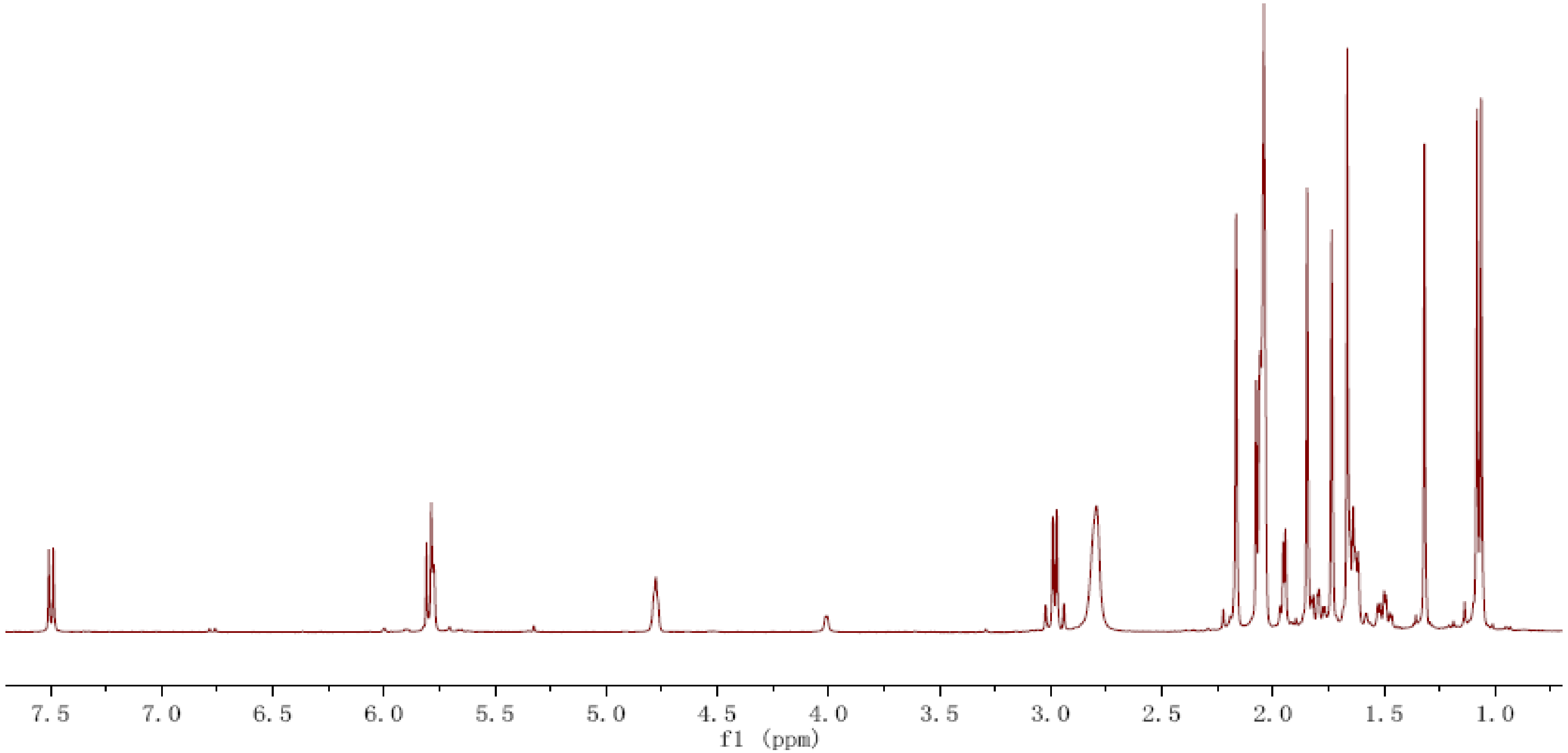

Figure S4. ^{13}C NMR Spectrum of Brevione I (**2**; 100 MHz, Acetone-d_6)

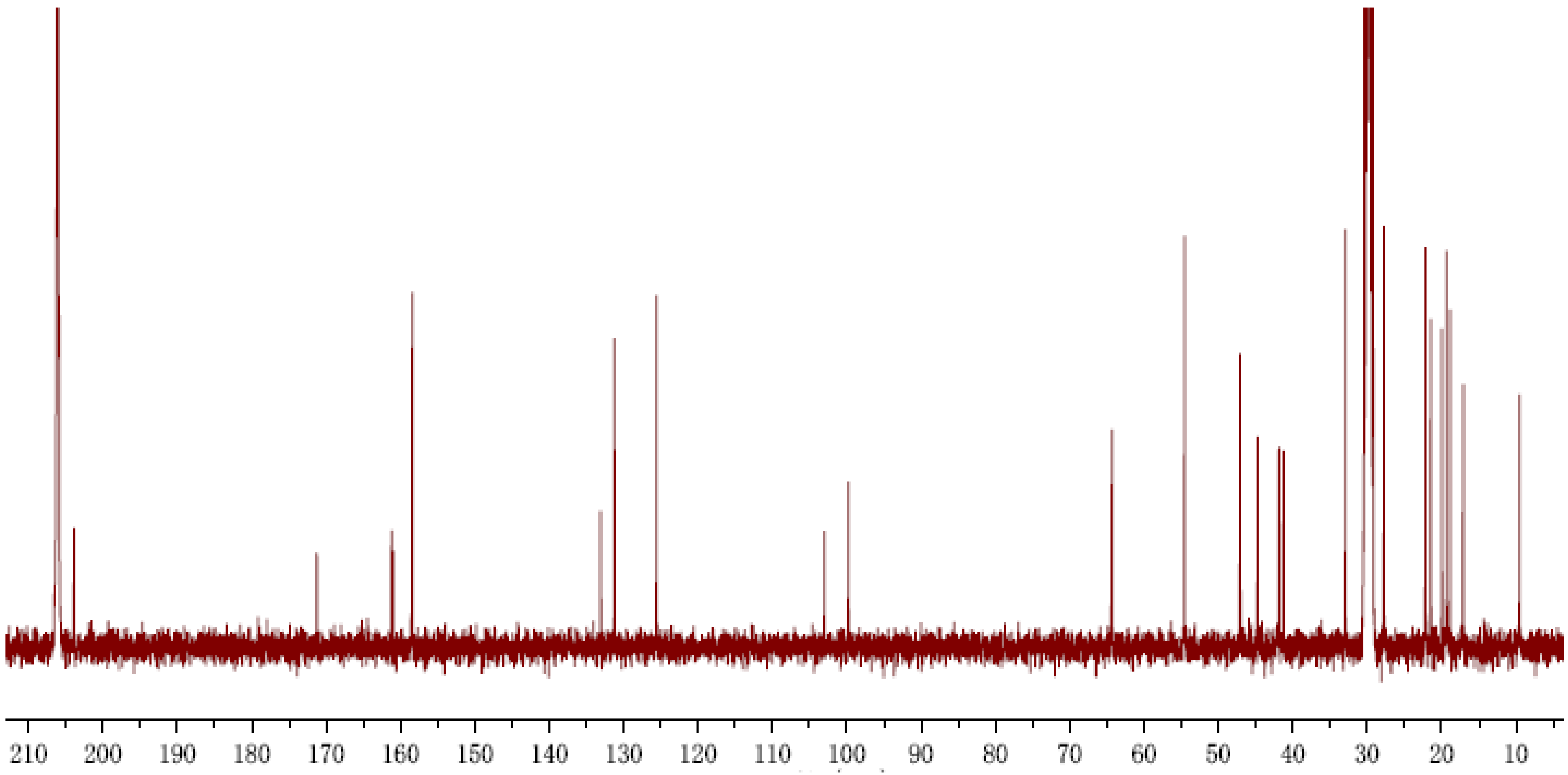

Figure S5. NOESY Spectrum of Brevione I (**2**; 600 MHz, Acetone-d_6)

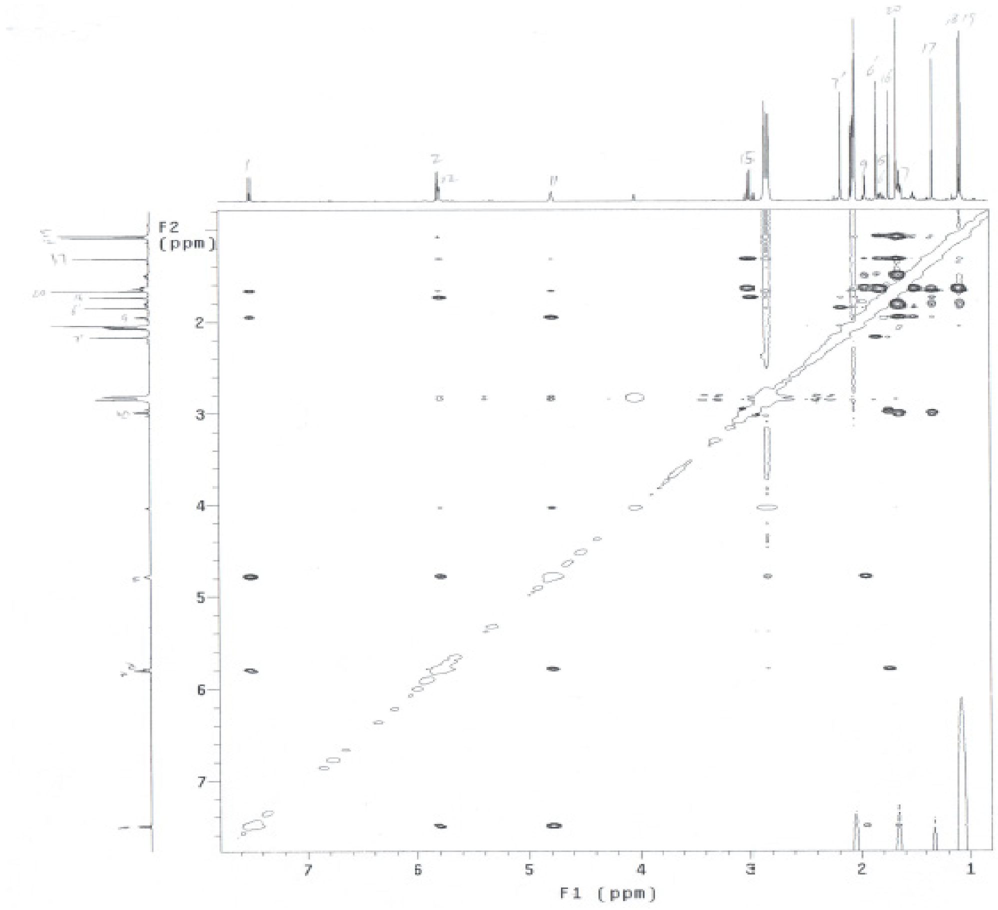

Figure S6. ^{1}H NMR Spectrum of Brevione J (**3**; 500 MHz, Acetone-d_6)

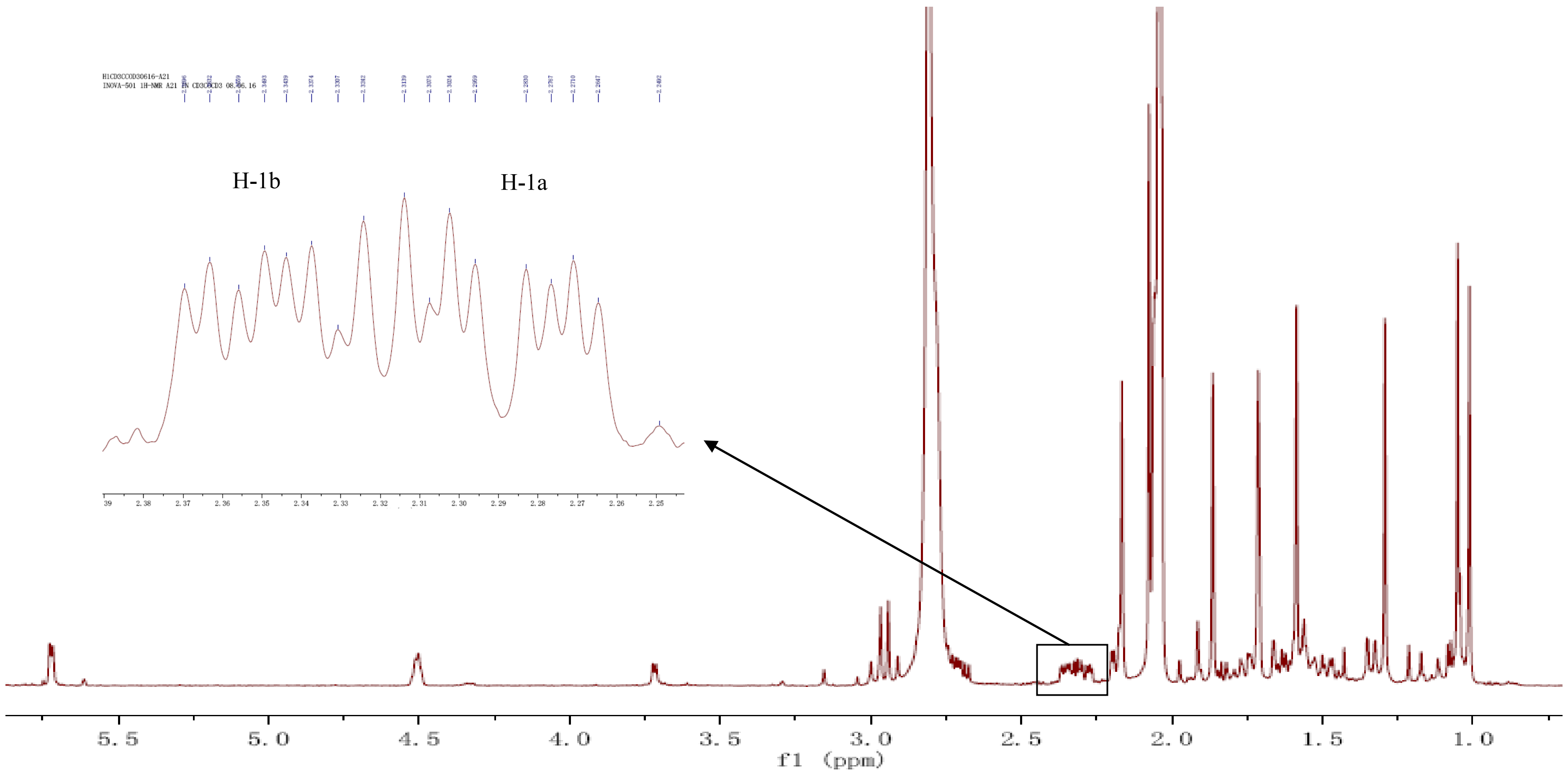

Figure S7. ^{13}C NMR Spectrum of Brevione J (**3**; 100 MHz, Acetone-d_6)

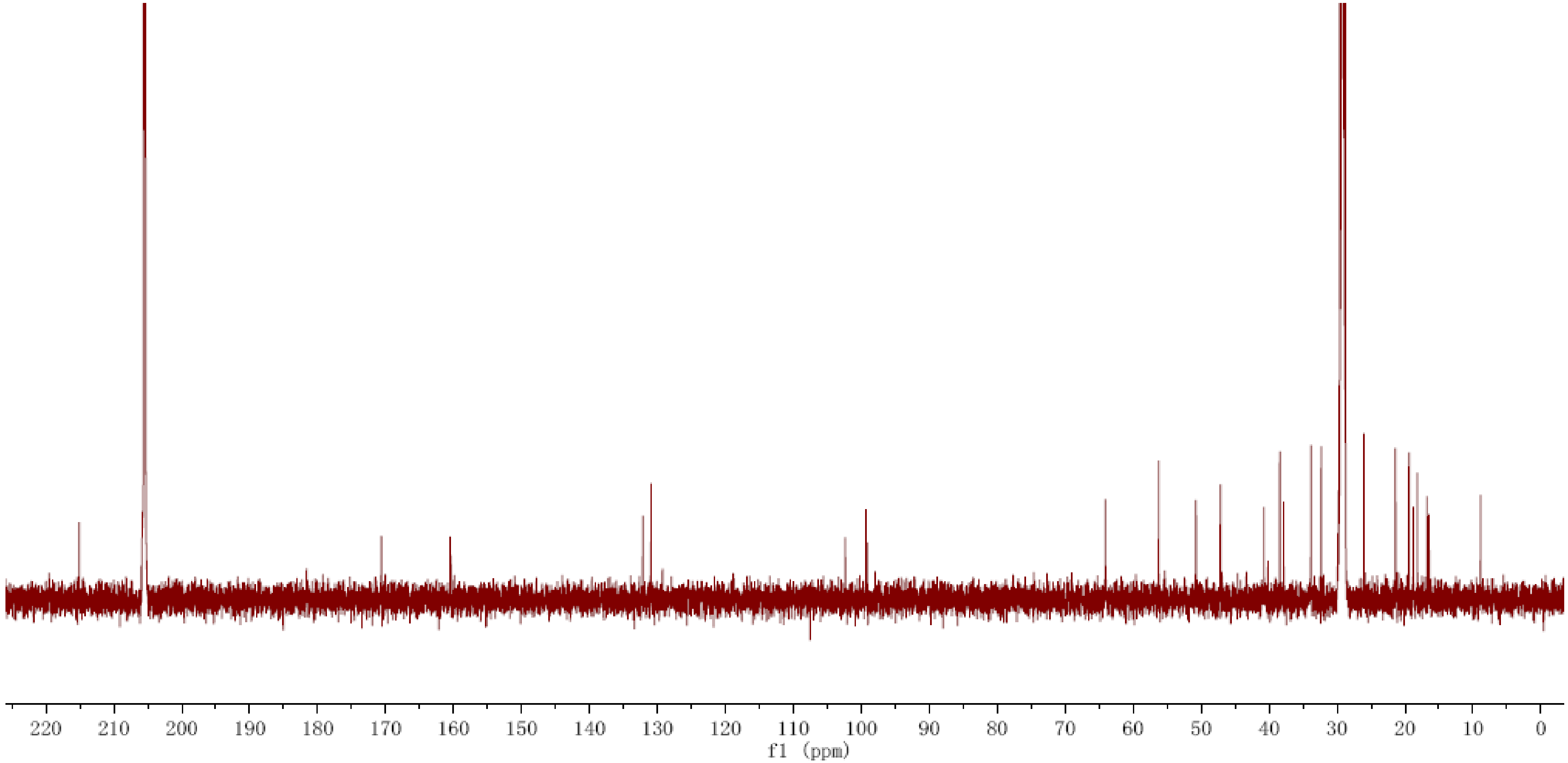

Figure S8. NOESY Spectrum of Brevione J (**3**; 600 MHz, Acetone-d_6)

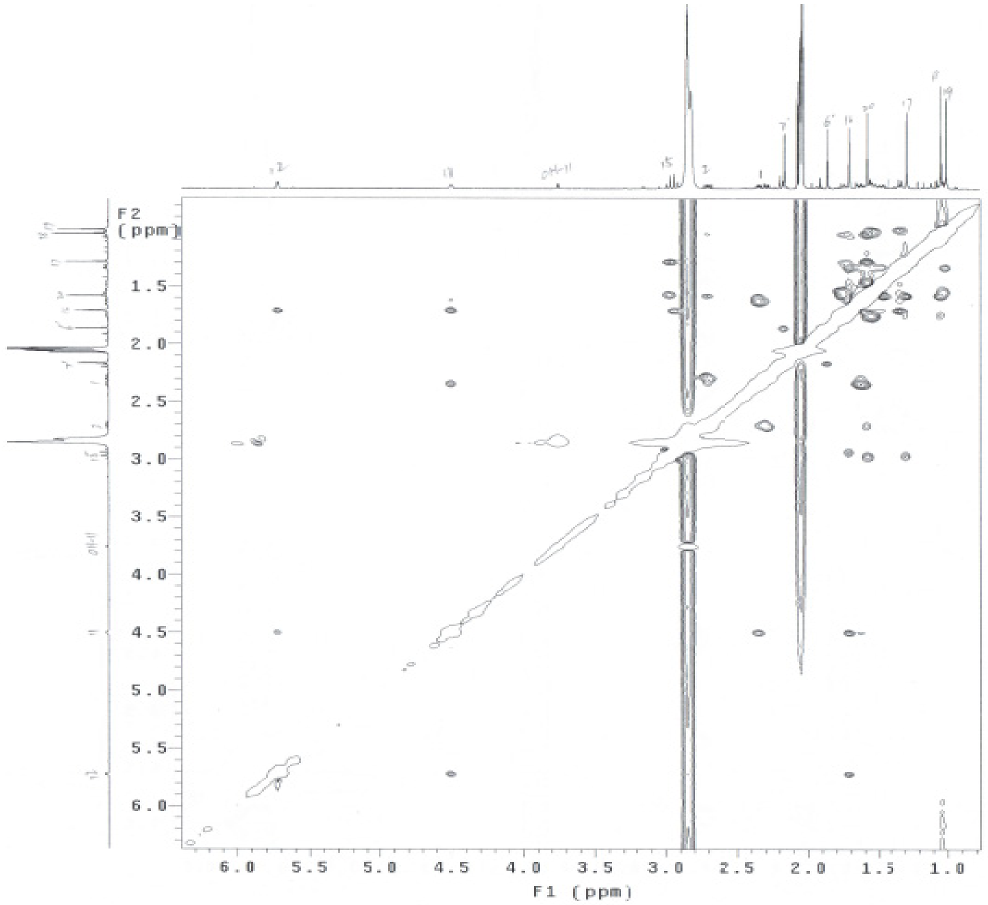

Figure S9. ^{1}H NMR Spectrum of Brevione K (**4**; 500 MHz, Acetone-d_6)

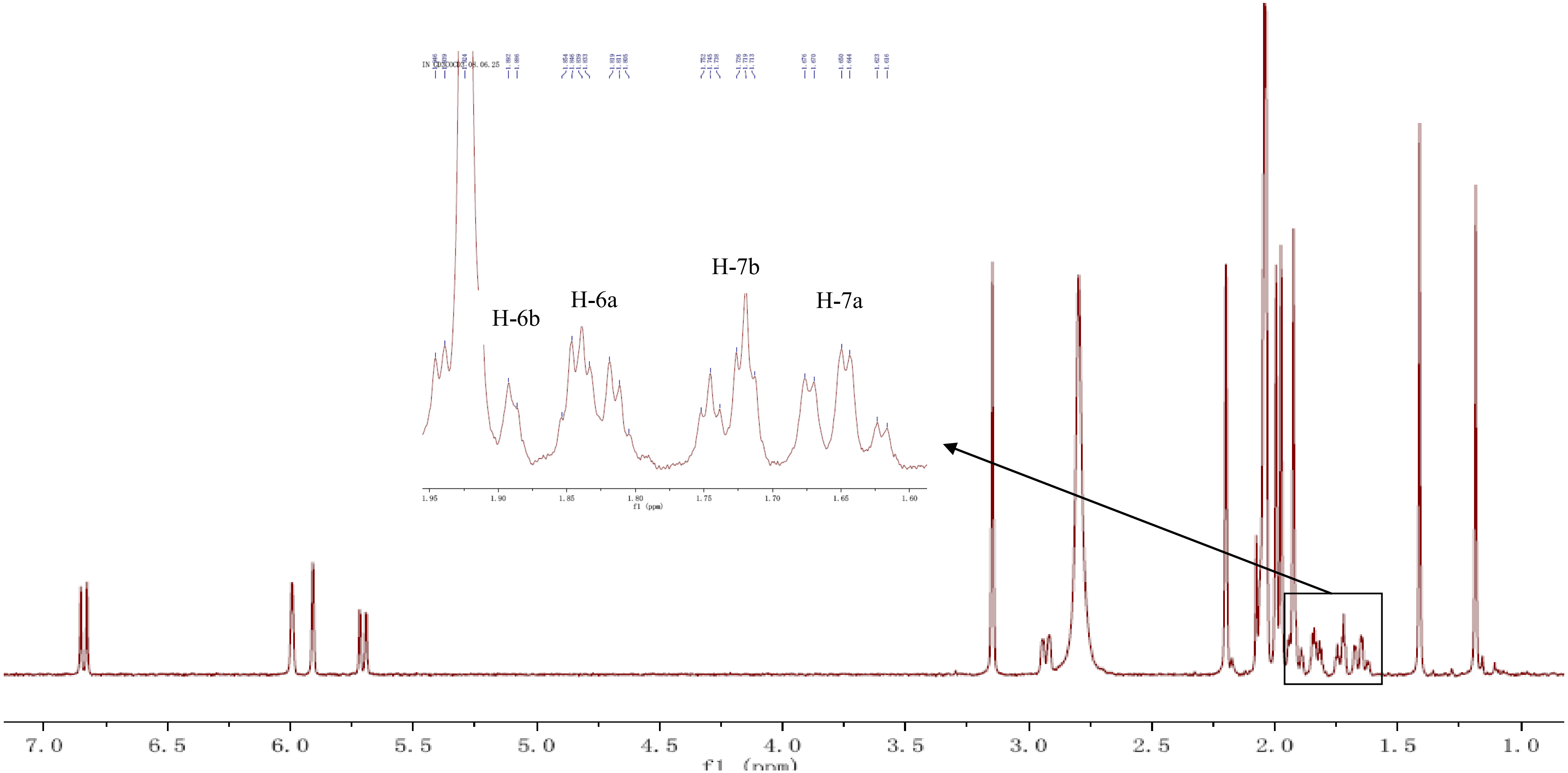

Figure S10. ^{13}C NMR Spectrum of Brevione K (**4**; 150 MHz, Acetone-d_6)

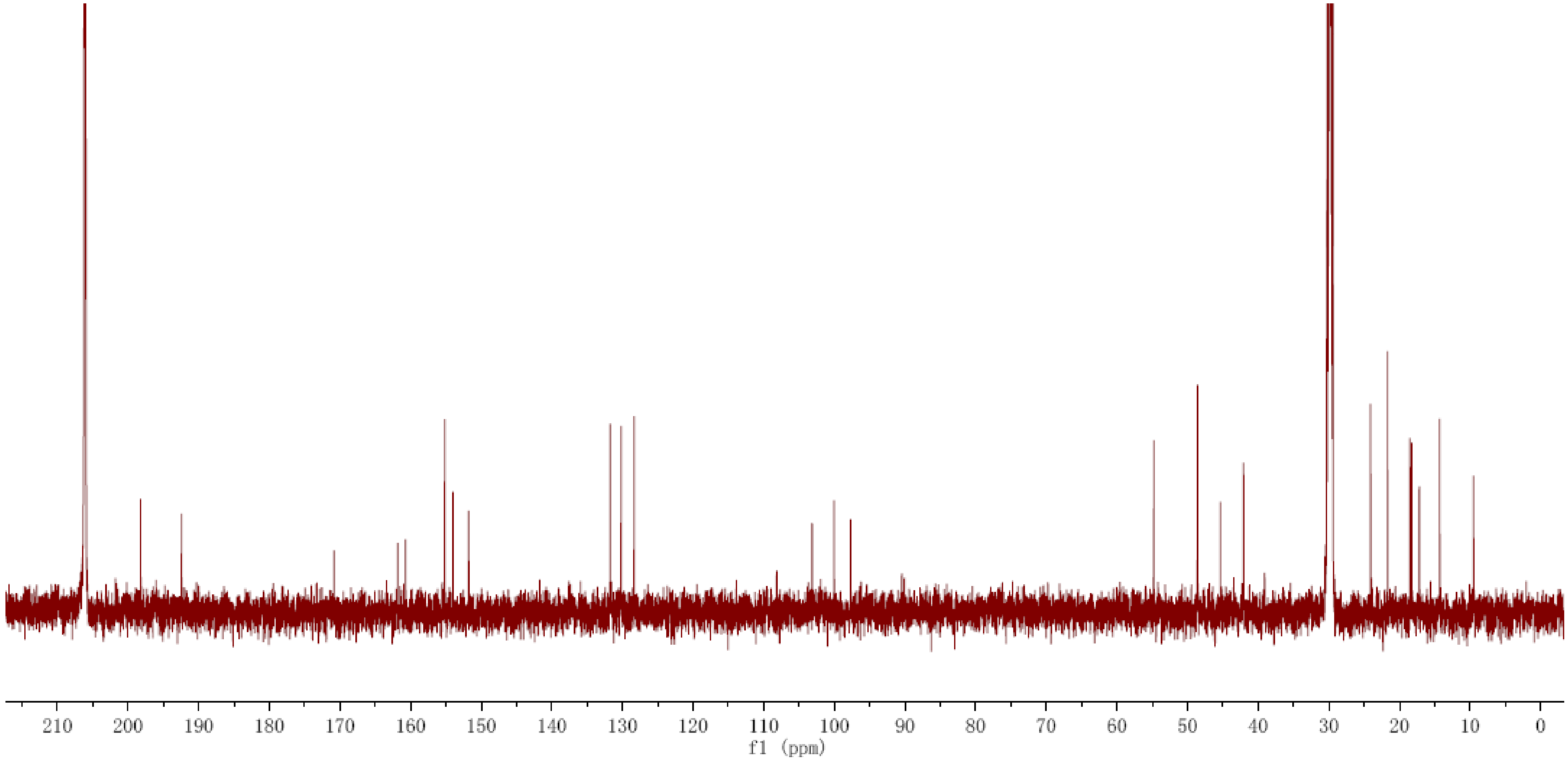

Figure S11. NOESY Spectrum of Brevione K (**4**; 600 MHz, Acetone-d_6)

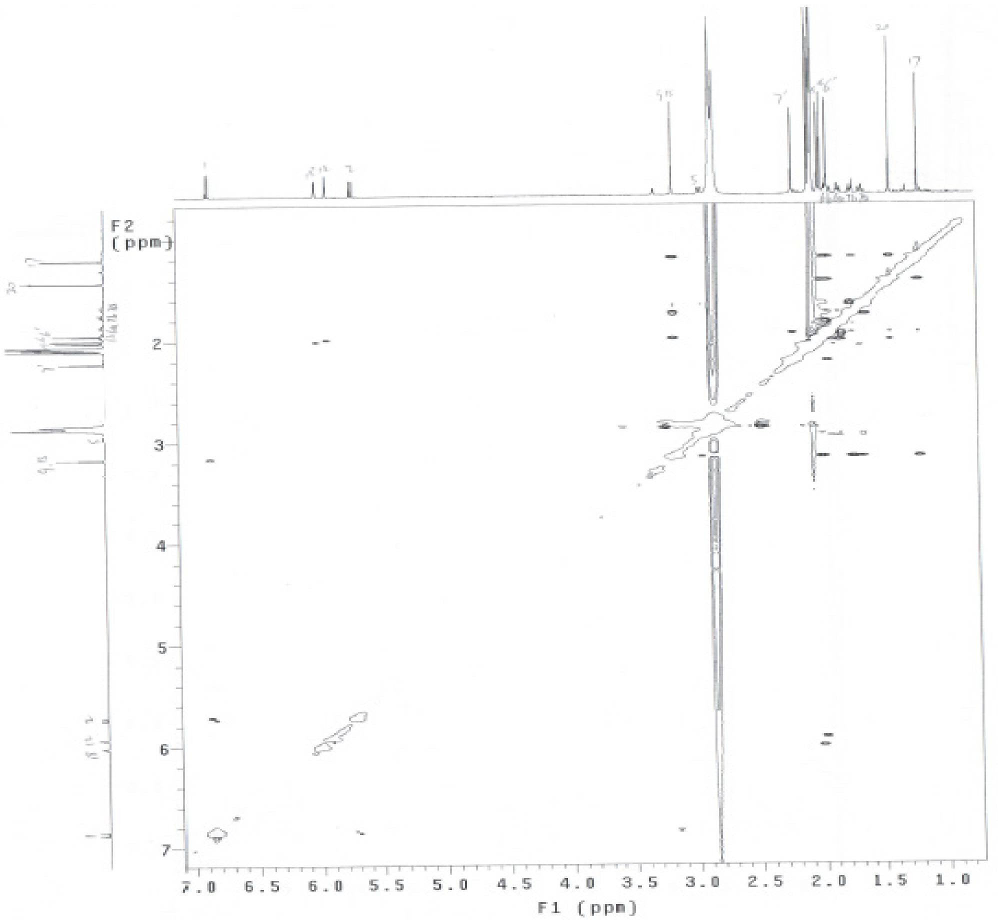

Supplement: Supplementary File 1: — ZIP-Document (ZIP, 299 KB) [file marinedrugs-10-00497-s001.zip › marinedrugs-14370-supplementary information.pdf]
